# Pressure Induced Changes in Adaptive Immune Function in Belugas (*Delphinapterus leucas*); Implications for Dive Physiology and Health

**DOI:** 10.3389/fphys.2016.00442

**Published:** 2016-09-30

**Authors:** Laura A. Thompson, Tracy A. Romano

**Affiliations:** Research and Veterinary Services, Mystic Aquarium, A Division of Sea Research Foundation Inc.Mystic, CT, USA

**Keywords:** marine mammal, immune function, proliferation, lymphocyte, PBMC, interleukin 2, diving, adaptive immunity

## Abstract

Increased pressure, associated with diving, can alter cell function through several mechanisms and has been shown to impact immune functions performed by peripheral blood mononuclear cells (PBMC) in humans. While marine mammals possess specific adaptations which protect them from dive related injury, it is unknown how their immune system is adapted to the challenges associated with diving. The purpose of this study was to measure PBMC activation (IL2R expression) and Concanavalin A induced lymphocyte proliferation (BrdU incorporation) in belugas following *in vitro* pressure exposures during baseline, Out of Water Examination (OWE) and capture/release conditions. Beluga blood samples (*n* = 4) were obtained from animals at the Mystic Aquarium and from free ranging animals in Alaska (*n* = 9). Human blood samples (*n* = 4) (Biological Specialty Corporation) were run for comparison. *In vivo* catecholamines and cortisol were measured in belugas to characterize the neuroendocrine response. Comparison of cellular responses between controls and pressure exposed cells, between conditions in belugas, between belugas and humans as well as between dive profiles, were run using mixed generalized linear models (α = 0.05). Cortisol was significantly higher in Bristol Bay belugas and OWE samples as compared with baseline for aquarium animals. Both IL2R expression and proliferation displayed significant pressure induced changes, and these responses varied between conditions in belugas. Both belugas and humans displayed increased IL2R expression, while lymphocyte proliferation decreased for aquarium animals and increased for humans and Bristol Bay belugas. Results suggest beluga PBMC function is altered during diving and changes may represent dive adaptation as the response differs from humans, a non-dive adapted mammal. In addition, characteristics of a dive (i.e., duration, depth) as well as neuroendocrine activity can alter the response of beluga cells, potentially impacting the ability of animals to fight infection or avoid dive related pathologies.

## Introduction

During diving, marine mammals must contend with several environmental and physiological challenges, including changes in pressure, against which they have developed specific physiological and behavioral adaptations. While a major concern with changes in pressure is the effect on air filled spaces, biological systems can also be affected at the cellular level. Inappropriate or impaired function of immune cells may lead to the development of infection if there is failure to mount a response or the development of autoimmune disease and self-damage if augmented responses occur. Several immune functions performed by macrophages and lymphocytes have been found to be sensitive to changes in pressure (Hallenbeck and Andersen, 1982), including antigen processing and the production of immunoglobulins. Macdonald (1982) reports that procession of the cell cycle is inhibited by increased pressures. This would impact the process of proliferation and the production of clones with specific antigen recognition capabilities (Murphy et al., 2008). In addition, denaturation of membrane associated proteins, including antigen receptors (Macdonald, 1982; Kato and Hayashi, 1999) may lead to decreased antigen binding and thus decreased immune responses. T cell activation relies on binding and clustering of T cell receptors (TCR's) and co-stimulatory molecules in the membrane (Germain, 1997; Quintana et al., 2005; Murphy et al., 2008) and so changes in membrane ordering could potentially lead to unwanted cell activation. As evidence of dive-induced changes in immune function, human divers, as compared to non-divers, show increased incidents of certain infections such as dermatitis and external ear infections (Edmonds and Shilling, 1984). Dive-related changes in immune function also play an important role in the development of decompression sickness (Ward et al., 1987; Barack and Katz, 2005).

Despite the extensive research concerning physiological and behavioral dive adaptations in marine mammals, very little is known about cellular responses to the challenges associated with diving, including changes in pressure. A prior study in our laboratory measured granulocyte activation, and granulocyte and monocyte phagocytosis in belugas in response to changes in pressure (Thompson and Romano, 2015). The data showed general decreases in phagocytosis immediately following pressure exposures, which recover to control levels post-decompression. The response of cells however, varied with characteristics of the dive such as depth and duration. Such unexpected alterations in immune function highlight the need to better understand the relationship between dive behavior, immune function and marine mammal health.

There are however, many factors that can affect immune function including exposure to stressors; loosely defined as stimuli that may impact homeostasis. Following perception of a stressor, behavioral, and physiological adjustments are made in order to deal with the threat, maintain homeostasis and adapt to different situations. The immediate purpose of this response is to promote survival, supporting vital functions over those which may be non-critical (Romero and Butler, 2007; Breuner et al., 2008). However, when these responses are extreme or become prolonged (i.e., chronic stress) negative effects on individual health may occur (Selye, 1936; Romero et al., 2009).

The neuro-endocrine response to a stressor is facilitated by activity of the sympathetic nervous system and hypothalamic pituitary adrenal axis resulting in the release of catecholamines (e.g., epinephrine and norepinephrine) and glucocorticoids (e.g., cortisol) from the adrenal glands. These hormones can exert influence on cell function by binding to specific receptors in the cell membrane (Madden et al., 1995; McEwen et al., 1997; Padgett and Glaser, 2003). In addition, norepinephrine serves as a neurotransmitter, effecting cells within close proximity to nerve endings (Padgett and Glaser, 2003). A direct anatomical link between the nervous and immune system has been described in cetaceans with functional data illustrating similar mechanisms (Romano, 1993; Romano et al., 1994, 2002).

The effects of a stress response on immune function are dependent on many factors including which receptors are bound, cell type, activation state of the cell, and the stimulus of immune responses (Madden et al., 1995; Madden, 2003) as well as the magnitude and duration of exposure (Dabhar, 2009; Martin, 2009). The act of diving itself can also initiate a stress response, either through physical stimulus of compression or, in the case of human divers, psychologically. The intensity of this stress response plays a role in determining the extent to which diving impacts immune function in humans (Philp, 1974). For example, more seasoned divers may experience less dive-related anxiety (Biersner and Larocco, 1987) and thus lesser changes in immune function. Similarly, the consequences of these changes in immune function may be either positive or negative. Enhanced immune activity may promote wound healing, for example, but suppression of immune activity may reduce the occurrence of autoimmune damage (Dabhar, 2009). However, if suppression occurs for too long, an individual may become susceptible to disease.

If humans experience a dive-related stress response leading to neuro-endocrine modulation of immune cell function, it is possible that neuro-endocrine activity can impact the ability of marine mammal cells to function during diving. In recent decades, the occurrence of gas bubbles and injury in stranded and by-caught marine mammals resembling decompression sickness seen in humans, have raised concern over the impacts of anthropogenic stressors on marine mammal health. It is unknown however, how marine mammal lymphocytes respond to the challenges of diving during “normal” conditions, and whether neuroendocrine responses to anthropogenic activity might affect this response.

This work was initiated in order to investigate the relationship between peripheral blood mononuclear cell (PBMC) activity, diving and stressors in belugas (*Delphinapterus leucas*). Belugas are a mid-size odontocete with a wide range of diving behaviors and deep diving capabilities (Hedrick and Duffield, 1991; Heide-Jorgensen et al., 1998; Martin et al., 1998; Martin and Smith, 1999; Suydam et al., 2001). Ultimately this work aims to describe the functional response of beluga PBMC's to changes in pressure under baseline and stressor conditions, as compared with humans. Specifically, the aims of this work were (1) to measure T lymphocyte proliferation and PBMC activation [through expression of the IL2 receptor (IL2R)] in belugas in response to simulated dive excursions *in vitro* (2) to compare the response of beluga PBMC's to the response measured in humans and 3) to evaluate the combined effects of pressure and an additional stressor on beluga PBMC activity *in vitro*. It was hypothesized that beluga cells would continue functioning at control levels following pressure exposures during baseline conditions, while human cells would show inhibition of function. Stressor conditions in belugas were expected to result in decreased measures of PBMC activity following pressure exposures, similar to that observed in humans.

## Methods

Baseline blood samples were obtained using positive behavioral reinforcement from four belugas resident at the Mystic Aquarium, Mystic, CT. Samples were also obtained from aquarium belugas at the conclusion of a 30 min Out of Water Examination (OWE, *n* = 3), which has previously been shown to result in changes in adrenocorticotropic hormone (ACTH) and cortisol (Schmitt et al., 2010). Samples were drawn from either the ventral or dorsal aspect of the flukes and collection from aquarium belugas was conducted under Mystic Aquarium IACUC protocol #110001 and UConn IACUC reciprocation #R12-002. In addition, blood samples were obtained from belugas in the Nushagak and Wood River areas of Bristol Bay, AK (*n* = 9) in accordance with NMFS Marine Mammal Research Permit No. 14245 and ADF&G permit No. 14610. For sampling in Bristol Bay, belugas are removed from an initial entanglement and restrained in shallow water for blood collection from the flukes. Human samples (*n* = 4) were purchased from Biological Specialty Corporation Inc., for comparison with belugas.

For cell function assays, whole blood samples were collected in 10 ml sodium heparin vacutainers and placed on ice for transfer to lab. Blood tubes were centrifuged for 10 min at 2000 × g and 10°C. Plasma was removed and 1 ml aliquots were stored in Sarstedt™ tubes at −80°C for hormone analysis. The white blood cell buffy coat was aliquoted in sterile cryovials and mixed with an equal volume of freezing media (90% fetal bovine serum and 10% DMSO). Buffy coats were stored at −80°C for 24 h and transferred to liquid nitrogen for storage until assayed. Samples collected from Bristol Bay animals were initially processed in the field and plasma and buffy coat samples were frozen immediately and shipped back to Mystic, CT in liquid nitrogen dry shippers.

### Simulated dives

Simulated dives were carried out by adding 4 ml of cell suspension to a stainless steel pressure chamber, and overlaid with a small layer of mineral oil to reach the desired pressure (Thompson and Romano, 2015). Two pressures were targeted in order to represent both an extreme dive [2000 psi (1360 m)] and a deep dive [1000 psi (680 m)] within the dive repertoire of belugas. Compression and decompression was either gradual (G) occurring over a period of 2 min, or rapid (R) occurring within 15 s. To encompass durations representative of the majority of beluga dives as well as extreme dives, pressure exposures lasted for 30 min, 5 min, or two repeated 5 min periods with a 1 min rest interval. Due to sample limitations, OWE and Bristol Bay samples were only exposed to 2000 psi with gradual compression and decompression (2000G).

### Interleukin 2 receptor (IL2R) expression

Archived white blood cell samples were thawed quickly at 37°C and washed twice with RPMI 1640. Final pellets were brought to a volume of 3 ml in RPMI 1640. Peripheral blood mononuclear cells (PBMC), including lymphocytes and monocytes, were isolated in the sample by Ficoll density gradient. Cell pellets, resuspended in 3 ml of RPMI 1640, were carefully overlaid on 3 ml of sterile histopaque 1077 and centrifuged for 30 min at 400 × g and 20°C. The separated mononuclear cell layer was then carefully removed and washed twice in cold Hank's Balanced Salt Solution (HBSS). Cell counts were obtained using Trypan blue staining (viability >95%) and sample volumes were adjusted to 2 × 10^6^ cells ml^−1^ with PBS. Two hundred microliter of sample were set aside for controls. Remaining sample was adjusted to a volume of 4 ml for simulated dive exposures. Following decompression, cells were recounted and volume re-adjusted to a concentration of 2 × 10^6^ cells ml^−1^ in PBS.

A human IL2 biotinylated fluorokine kit was used to assess lymphocyte activation (R and D systems, Minneapolis, MN). Specificity testing as per manufacturer's instructions, as well as Con A stimulation tests were carried out on beluga samples. Twenty five microliter of cell suspensions were aliquoted into Falcon™ tubes, and 10 μl of biotinylated IL2 cytokine was added. Negative controls received 10 μl of biotinylated soybean trypsin inhibitor (negative control). All tubes were incubated for 60 min at 4°C after which 10 μl of avidin FITC were added. Tubes were then incubated for a further 30 min in the dark at 4°C. Tubes were washed twice with 2 ml of RDF1™ wash buffer and final cell pellets were re-suspended in 250 μl of 1% paraformaldehyde until flow cytometric analysis.

### Flow cytometry

Samples were read using an LSR flow cytometer (BD Biosciences, San Jose, CA). For all assays; samples were read within 24 h following addition of paraformaldehyde. PBMC, including lymphocytes and monocytes, were gated using forward and side scatter plots from controls containing only cells. The FITC was read at 518 nm in the FL1 channel. For IL2R expression, the mean intensity of the fluorescence (MFI) expressed by PBMC's was collected. This measure is reflective of the relative amount of expression per cell i.e., increased MFI reflects an up-regulation in expression. Ten thousand events within the gated population were targeted.

### Lymphocyte proliferation

A colorimetric 5-bromo-2′-deoxyuridine (BrdU) incorporation ELISA kit was purchased from Roche Applied Sciences (Indianapolis, IN) and used to measure Concanavalin A (Con A) induced T lymphocyte proliferation. Buffy coats were thawed quickly at 37°C and washed twice with RPMI 1640 (centrifuged for 5 min at 220 × g and 20°C). PBMC's were isolated via Ficoll density gradient as described above. The separated cell layer was carefully removed and washed twice in media (RPMI 1640 with 0.1 μM non-essential amino acids, 100 units ml^−1^ penicillin, 100 μg ml^−1^ streptomycin, 0.292 mg ml^−1^ 1-glutamine, 1% 100 mM sodium pyruvate, 1% 1 M hepes, 10% FBS, 1% 0.01 M 2-mercaptoethanol).

Cell counts were obtained using Trypan blue exclusion (viability >95%) and sample volume was adjusted to reach a target of 10^6^ cells ml^−1^. Control cells were set aside and the remaining cells were brought to 4 ml for simulated dive exposures. Following each dive, cells were recounted and final volume readjusted to reach 10^6^ cells ml^−1^.

One hundred microliters of cell suspensions were aliquoted into BD Falcon™ 5 ml polystyrene round bottom tubes (BD Biosciences, San Jose, CA) and 100 μl of Con A working stock (5 μg ml^−1^) was added for a final Con A concentration of 2.5 μg ml^−1^, which had previously been determined in our laboratory to be optimal for belugas. Controls did not receive any Con A. Plates were incubated for 72 h in a 5% CO_2_ incubator at 20°C. At 72 h, 20 μl of BrdU was added to sample wells, as well as to BrdU controls, and plates were returned to the incubator for an additional 18 h. After a total incubation of 90 h, wells were pipetted to break up aggregates and plates were centrifuged for 10 min at 300 × g. Wells were then emptied and plates dried under a hair dryer for 15 min. Dried plates were then sealed in Ziploc bags and stored at 4°C until development. All plates were developed according to kit instructions within 1 week of drying.

Two hundred microliters of FixDenat™ solution were added to each well and incubated for 30 min at room temperature. Solution was emptied from wells and 100 μl of anti-BrdU-POD (peroxidase conjugated monoclonal Fab fragments) were then added to each well. After a further 90 min incubation, wells were emptied and washed three times with media. One hundred microliters of substrate solution (tetramethyl-benzidine) were then added and color was allowed to develop for 30 min. Twenty five microliters of 1 M H_2_SO_4_ were added to stop the reaction. Absorbance was read at 450 nm using an EL800 microplate reader (BioTek, Winooski, VT). Stimulation indices were calculated for each sample as the ratio of the optical density (OD) of stimulated cells to the OD of the control cells with BrdU but no mitogen.

### Hormone analysis

One ml of archived plasma was shipped to the AHDC Endocrinological Lab at Cornell University (Ithaca, NY) for cortisol analysis using the Immulite® system (Schmitt et al., 2010; Spoon and Romano, 2012; Schwake et al., 2014). Catecholamines were measured at the Mystic Aquarium using a Waters (Milford, MA) High Performance Liquid Chromatography system (1515 isocratic pump, 717 auto sampler) with 2465 electrochemical detection. Hormones were extracted using 50 mg of acid washed alumina (BioRad Cat. 195-6055) and an internal standard was added to each sample. Details of this methodology are reported in Thompson and Romano (2015).

### Statistics

Hormone levels and control values of immune function were compared between baseline, OWE conditions and Bristol Bay animals using a mixed generalized linear model or ANOVA. All measures of immune function following pressure exposures were normalized over control values for all comparisons. Mixed effects generalized linear models with repeated measures were used to evaluate (1) significant pressure induced changes in cell function as compared with non-pressure exposed cells, (2) significant differences in the response of cells to different dive characteristics, i.e., duration or depth, (3) significant differences in the response of beluga immune cells between baseline and OWE conditions, and between aquarium and Bristol Bay belugas, and (4) significant differences in the response of beluga cells as compared with humans. Significance was determined at α = 0.05. Due to small sample sizes however *p* < 0.1 are also reported as observed patterns.

## Results

### Effects of pressure on baseline beluga samples

#### Effects of 2000 psi with 2 min compression/decompression (2000G) on lymphocyte activation and proliferation

Results of pressure exposures on immune function are summarized in Table 1. Baseline beluga samples displayed a significant increase in IL2R expression following both the 5 min (*p* = 0.031) and repeated 5 min (*p* = 0.005) exposures to 2000 psi with 2 min of compression and decompression. Despite this increase in expression suggesting cell activation, baseline samples displayed significant decreases in proliferative activity following the 30 min (*p* < 0.001) and repeated 5 min (*p* = 0.032) exposures.

**Table 1:** **Summary of results for IL2 expression and lymphocyte proliferation following simulated pressure exposures for baseline beluga samples**.

**Pressure**	**Duration**	**IL2R expression**	**Lymphocyte proliferation**
2000G	30 min		*p* < 0.001
	5 min	*p* = 0.031	
	2 × 5 min	*p* = 0.005	*p* = 0.032
2000R	30 min	*p* = 0.019	*p* < 0.001
	5 min	*p* = 0.007	*p* = 0.013
	2 × 5 min	*p* = 0.031	
1000G	30 min	*p* < 0.001	*p* < 0.001
	5 min	*p* < 0.001	No data
	2 × 5 min	*p* < 0.001	No data
1000R	30 min	*p* = 0.001	*p* = 0.032
	5 min		No data
	2 × 5 min		No data

*Exposures with gradual compression and decompression occurring over 2 min are represented by “G”, while exposures with rapid compression and decompression occurring over 15 s are represented by “R”. Pressure induced changes in immune functions are indicated by shading; no color, no change, dark gray, decreased function. Light gray, increased function*.

#### Effects of 2000 psi with 15 s compression/decompression (2000R) on lymphocyte activation and proliferation

General patterns of increased expression of IL2R were observed for baseline beluga samples following all exposures to 2000 psi with rapid compression and decompression (Table 1; 30 min, *p* = 0.019; 5 min, *p* = 0.007; 2 × 5 min, *p* = 0.031). In contrast, significant decreases in proliferation were detected for baseline conditions in belugas following the 30 min (*p* < 0.001) and single 5 min (*p* = 0.013) exposures (Table 1).

#### Effects of 1000 psi with 2 min compression/decompression (1000G) on lymphocyte activation and proliferation

The expression of IL2R increased significantly in baseline beluga samples following all duration exposures to 1000 psi with 2 min of compression and decompression (Table 1; *p* < 0.001 for all exposures). Samples for proliferation experiments were only available for the 30 min exposures, and a significant decrease was detected (Table 1; *p* < 0.001).

#### Effects of 1000 psi with 15 s compression/decompression (1000R) on lymphocyte activation and proliferation

Significant pressure induced changes in MFI for IL2R expression were detected only during the 30 min exposure, for which belugas displayed a significant increase in expression (Table 1; *p* = 0.001). Samples were only available for 30 min exposures to 1000 psi with rapid compression and decompression for proliferation experiments. A decrease in proliferation was measured following this exposure (Table 1; *p* = 0.032).

### Comparative effects of pressure between dive exposures

#### IL2R expression

**Figure 1:**
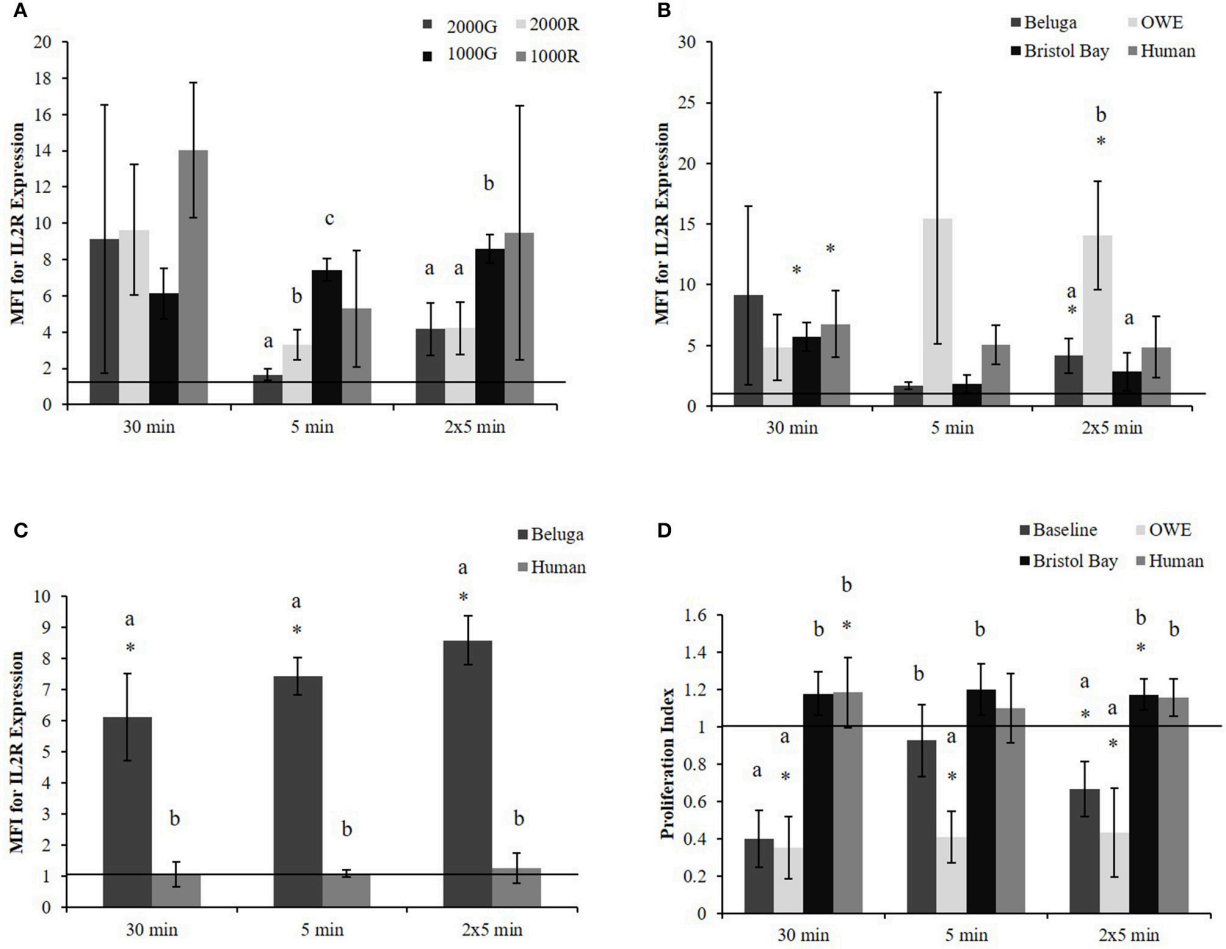
**PBMC MFI for IL2R expression in aquarium belugas**. **(A)** During baseline conditions (*n* = 4) following all pressure exposures; **(B)** in aquarium belugas during baseline (*n* = 4), and OWE (*n* = 3) conditions, in belugas from Bristol Bay, AK (*n* = 9) and humans (*n* = 4) following exposures to 2000G; **(C)** in aquarium belugas during baseline (*n* = 4) conditions vs. humans (*n* = 4) following exposures to 1000G; **(D)** Lymphocyte proliferation in aquarium belugas during baseline (*n* = 4) and OWE conditions (*n* = 3), in belugas from Bristol Bay, AK (*n* = 9) and humans (*n* = 4) following exposures to 2000G. Data are normalized to controls (represented by the solid line at 1) and presented as the mean ± SE. Values greater than 1 indicate increased function, and values less than 1 indicate decreased function following pressure exposures. Within each duration, significant differences between exposure profiles (e.g., 2000G vs. 2000R), between conditions in belugas (e.g., baseline vs. OWE) or between belugas and humans are indicated with letters (*p* < 0.05). Significant differences from controls are indicated with an asterisk *(*p* < 0.05).

Pressure induced changes in the average expression of IL2R per cell were significantly different between dive profiles for the 5 min and repeated 5 min duration exposures. For both durations (Figure 1A), exposures to 1000G resulted in larger changes than either 2000G (5 min, *p* < 0.001; 2 × 5 min, *p* = 0.003) or 2000R (5 min, *p* < 0.001; 2 × 5 min, *p* = 0.002). In addition, exposure to 2000G resulted in significantly smaller changes in IL2R expression than exposure to 2000R for the single 5 min exposure (*p* = 0.036). A greater response to 1000R as compared with 1000G was also observed for the 30 min exposures (Figure 1A; *p* = 0.064).

No significant differences were found between exposure duration for any dive profiles. However, results suggest single 5 min exposures result in smaller change in IL2R expression as compared with 30 min exposures for 1000R (*p* = 0.098) and 2000R (*p* = 0.064).

#### Lymphocyte proliferation

No significant differences in proliferation indices were detected between dive exposures, i.e., different pressures and rates of compression/decompression. Additionally no significant differences in proliferation indices were detected between pressure exposures of different durations.

### Effects of condition (baseline vs. OWE) on the response of beluga lymphocytes to pressure exposures

#### Hormones

Plasma hormone levels for baseline and OWE conditions in belugas have been reported previously (Thompson and Romano, 2015) and are presented here for comparison with Bristol Bay animals (Table 2).While epinephrine levels were below the detectable range of the HPLC protocol (<30 pg ml^−1^) for most baseline samples, epinephrine as well as norepinephrine were significantly higher in Bristol Bay belugas than both baseline and OWE conditions for aquarium belugas. Though significant differences were not detected for cortisol, Bristol Bay belugas also displayed higher plasma cortisol values than baseline conditions for aquarium animals.

**Table 2:** **Plasma hormone concentrations (mean ± SE) from Bristol Bay belugas compared with Baseline and OWE conditions in aquarium belugas (Baseline and OWE data originally presented in Thompson and Romano, 2015)**.

	**Epinephrine pg ml^−1^**	**Norepinephrine pg ml^−1^**	**Cortisol μg dl^−1^**
Bristol Bay *n* = 9	341.9 ± 48.68	1461.08 ± 240.48	6.43 ± 0.79
Baseline *n* = 4	ND^*^	662.96 ± 110.5^*^	1.57 ± 0.2
OWE *n* = 3	78.02 ± 43.4^*^	757.056 ± 81.4^*^	7.97 ± 1.2^**^

ND, not detectable. Significant differences from wild animals are indicated by an asterisk

(*). Significant differences from baseline are indicated by a double asterisk

(**). *p < 0.05*.

#### Effects of 2000 psi with 2 min compression/decompression (2000G) on beluga OWE and bristol bay beluga samples

Significant increases in the expression of IL2R (Figure 1B) were detected for Bristol Bay samples following the 30 min exposure to 2000G (*p* < 0.001) as well as in OWE samples following the repeated 5 min exposures (*p* = 0.030).

OWE samples displayed decreased proliferation following the 30 min (*p* < 0.001) and repeated 5 min exposures (*p* = 0.024). OWE samples also displayed pressure induced decreased proliferation for the 5 min exposure (*p* < 0.001). In contrast, animals from Bristol Bay displayed a significant increase in proliferation following the repeated 5 min exposures (*p* = 0.045).

#### Comparative effects of pressure on beluga and human samples

The increase observed in IL2R expression following exposure to 2000G for OWE samples was significantly larger than the change observed in both baseline conditions for aquarium belugas (*p* = 0.038) and in Bristol Bay animals (*p* = 0.022) for the repeated 5 min exposures (Figure 1B). Significant increases in IL2R expression measured in baseline conditions in belugas for exposures to 1000G were significantly larger than the change observed in humans (Figure 1C).

Pressure induced changes in proliferation were also significantly different between conditions for each duration exposure. In general, baseline beluga samples and OWE samples showed decrease proliferation while Bristol Bay samples and humans showed increased proliferation (Figure 1D). For both the 30 min (*p* < 0.001) and repeated 5 min exposures (*p* = 0.014) the response of beluga cells during baseline conditions was significantly larger than humans. In addition humans displayed a significantly smaller response as compared with OWE conditions in belugas for all durations (30 min, *p* < 0.001; 5 min, *p* = 0.008; 2 × 5 min, *p* = 0.012). Bristol Bay animals displayed significantly smaller responses than aquarium animal baseline samples following the 30 min (*p* < 0.001) and repeated 5 min exposures (*p* = 0.008), and OWE samples for all duration exposures (30 min, *p* < 0.001; 5 min, *p* = 0.001; 2 × 5 min, *p* = 0.009). In addition, aquarium belugas displayed significantly larger responses during OWE conditions as compared with baseline, for the single 5 min exposure (*p* = 0.042).

## Discussion

One of the natural challenges associated with diving is changing pressures, the effects of which can lead to cellular dysfunction in the central nervous system (Bennett, 1982; Macdonald, 1982), bone necrosis (McCallum and Harrison, 1982) and disease in humans. Few studies have investigated the effects of pressure on cell function in marine mammals, for which diving is an integral behavior for survival. Field (2000) found that elephant seal platelets responded differently than human platelets to *in vitro* increased pressure as well as decreased temperature. Cholesterol content of the platelet membranes was noted to be one mechanism of adaptation in the elephant seal. Castellini et al. (2001) found that following *in vitro* exposure to pressure, glucose uptake and production of lactate by red blood cells differed between terrestrial mammals, shallow diving marine mammals and deep diving marine mammal species. In addition, granulocyte and monocyte phagocytosis in belugas also appears to be altered when cells are exposed to high pressures (Thompson and Romano, 2015). This study now provides the first investigation of the effects of pressure on PBMC function in belugas, a dive adapted mammal as compared with humans, a non-dive adapted mammal.

Following *in vitro* pressure exposures with beluga PBMC's, there was an overall pattern of increase for IL2R expression. Humans seemed to display smaller changes than baseline conditions in belugas for exposures to 1000 psi, and larger changes than baseline belugas for exposure to 2000 psi. In contrast, proliferation in baseline belugas samples decreased in most cases, and showed no significant change in humans.

Increases observed in measures of IL2R expression on human PBMC suggest that changes in pressure result in activation of human cells. A possible mechanism for this result is through altering membrane characteristics. T cell activation requires binding of the T cell receptor and an associated cluster of co-stimulatory receptors. The receptors exist within the membrane as parts of mobile lipid rafts which aggregate as part of the activation signal. Aggregation of these rafts has been reported to occur in response to changes in membrane fluidity at decreased temperatures (Magee et al., 2005). Increased pressure, like decreased temperature also has an ordering effect on cell membranes (Macdonald, 1982; Siebenaller and Garrett, 2002) and thus may have led to signaling and increased IL2R expression. Increased IL2R expression is also indicative of monocyte activation, playing important roles in regulating cell differentiation and effector functions (Herrmann et al., 1985; Espinoza-Delgado et al., 1995). IFN γ released, in this case by pressure-activated T lymphocytes, may have influenced monocyte activation and up-regulation of IL2R expression (Herrmann et al., 1985).

Interestingly this effect of increasing IL2R expression was also seen in beluga PBMC exposed to pressure. In some cases, particularly following exposures to 1000G, the changes observed for belugas were larger than that observed for humans. This pattern of increase differs from general decreases observed in granulocyte phagocytosis for belugas (Thompson and Romano, 2015), thus suggesting that different cell types with different functions respond differently to the effects of pressure. Additionally, while a decrease in immune function was hypothesized to be protective against the development of inflammatory damage associated with dive-related injury, activation of lymphocytes in belugas, may suggest that these cells are less involved in the development of such conditions. One role IL2R expression has in monocytes may be to regulate IL2 signaling in T lymphocytes (Herrmann et al., 1985; Toossi et al., 1990). Increased expression and binding within the monocyte population for example may mean less binding within the lymphocyte population thus preventing damaging lymphocyte responses; such as the development of auto-immune diseases and inflammation (Shimizu et al., 2002; Feske, 2007; Murphy et al., 2008).

Because this study showed an overall increase in IL2R expression following pressure exposures, suggesting cellular activation, it was expected that an increase in proliferative responses would follow. IL2 signaling is an important early step in proliferation for lymphocytes but does not induce proliferation among monocytes (Espinoza-Delgado et al., 1995). Decreased proliferative responses have been reported in human lymphocytes stimulated with phytoheamagglutinin following exposure to increased pressure (Macdonald, 1982) and for this study decreased proliferation following pressure exposures occurred in aquarium belugas, while no significant change was detected for humans.

Decreased proliferation can result from mechanical inhibition of cell division or altered ability of mitogen receptors to bind with the stimulus (Macdonald, 1982). Thus, even though results from this study suggest an activation of lymphocytes, it is possible that the pressures used (representing extreme or challenging dives in the repertoire of belugas) resulted in a change in membranes or receptors that prevented a proliferative response. Additionally, these results could suggest different sensitivity of early and later stages of PBMC activation to the effects of pressure.

In contrast humans displayed either an expected increase or no significant change in IL2 expression, but no significant change in proliferation indices. The difference in responses between species warrants further investigation and may reflect specific adaptations on beluga cells to cope with potential challenges of diving. Though beyond the capabilities of this work, it would be interesting to (1) repeat these studies at lower pressures or (2) determine if beluga cells are able to recover post-decompression. For example, beluga granulocytes showed decreased phagocytic activity immediately following pressure exposures, but these values returned to control levels after a further 20 min (Thompson and Romano, 2015).

In general, changes in both IL2R expression on PBMC's and lymphocyte proliferation were greater following the 30 min exposures than shorter exposures. The relationship of the change in IL2R expression between belugas and humans appeared to vary with characteristics of the dive (i.e., for exposures to 1000 psi belugas displayed larger changes, while for exposures to 2000 psi humans displayed larger changes). Belugas also displayed larger changes in IL2R expression following 1000 psi exposures as compared with 2000 psi exposures for dives with 2 min of compression and decompression. These comparisons may suggest there is some limitation to dive adaptation in these cells, and thus a trade-off between dive behavior (depth, duration) and maintaining health. In addition these results suggest that forced longer dives, perhaps as part of an avoidance response, could lead to unusual changes in PBMC activity. Alternatively, if the changes observed in baseline beluga samples represent protective adaptation then larger changes observed during longer dives may suggest plasticity in the response of PBMC, similar to that observed with other aspects of the dive response. For example, the degree to which bradycardia occurs has been reported to be related to dive duration in Weddell seals (Kooyman et al., 1980). If this is the case, there may be a range of dive behaviors within which the risk of disease or injury is minimal, and this risk may vary depending on the characteristics of a dive (i.e., depth, duration) or among deep and shallow diving species.

There were also some observable differences in the response of cells between conditions in belugas, suggesting that there is an intricate relationship between neuroendocrine activity, dive behavior and health in these animals. Previous work showed an OWE resulted in increased cortisol values in belugas (Schmitt et al., 2010). For this study, cortisol values were similar between OWE and belugas from Bristol Bay, AK. Bristol Bay animals, however, showed higher catecholamine values than all conditions in aquarium animals, which is not unexpected. Free range belugas are faced with many potential stressors, including boat noise and subsistence hunting. In addition, during live capture and release studies, these animals were followed, netted and restrained in shallow water for sampling and examination; a process which induces a physiological response (Ortiz and Worthy, 2000; Forney et al., 2002). Some differences were noted in control measures of immune function between conditions, with general patterns suggesting decreased control values for OWE conditions and Bristol Bay animals as compared with baseline conditions in aquarium belugas (data not shown). Bristol Bay belugas also displayed significantly lower indices of proliferation than OWE and baseline conditions for aquarium animals. These findings are not unexpected, as increased catecholamines and cortisol have been linked with decreased measures of immune function (Nieman et al., 1994; Qiu et al., 2005; Webster-Marketon and Glaser, 2008; Dabhar, 2009). As part of a healthy neuroendocrine response, this temporary decrease in immune activity may aid in the reallocation of resources (i.e., energy) to more high-priority functions (Segerstrom, 2007; Rauw, 2012).

Following pressure exposures, larger changes in IL2R expression for belugas were observed during OWE conditions as compared with baseline. While plasma cortisol was similar between OWE conditions and Bristol Bay samples, changes in IL2R expression were larger in OWE samples as compared with Bristol Bay animals following the single and repeated 5 min exposures. In addition, there was also a larger decrease in proliferation for OWE samples, similar to the drop in baseline samples, as compared to the small increased observed in samples from the Bristol Bay animals. Not only are the magnitudes of changes different but the direction of response is different between groups of belugas in this study. These differences may potentially reflect the intensity, duration, and type of neuroendocrine stimulus, as well as prior experience of aquarium vs. Bristol Bay belugas.

Free ranging animals from Bristol Bay will have had more opportunity to experience deep or varied dive patterns as compared with aquarium animals. Previous dive experience has been reported to refine dive capabilities and produce an acclimatization effect which reduces occurrence of decompression sickness (Ferretti and Costa, 2003; Lander et al., 2003; MacArthur et al., 2003; Su et al., 2004) and thus this experience may have resulted in cells from Bristol Bay belugas being less responsive to pressure exposures. The nature of the neuroendocrine stimulus also differs between Bristol Bay and aquarium belugas. The OWE was of shorter duration compared with the chase and restraint of Bristol Bay animals, which could also have a greater stressor load as they face challenges associated with foraging, predation and hunting.

Interestingly, changes in proliferation following pressure exposures in the Bristol Bay animals more closely resembled that measured in humans, as compared with aquarium animals. It is difficult to determine the implications of these differences on individual animal health, yet the comparison highlights the need to further examine the relationship between physiological status and immune responses during diving. While the neuro-endocrine response to handling may have played a role, no significant correlations were found between hormone values and changes in proliferation for any pressure exposures (data not shown), and so there may have been other factors impacting the response of cells from the Bristol Bay animals as compared with aquarium animals. For example, there can be an effect of diet on the membrane composition of lymphocytes between the two groups which can alter cell sensitivity to pressure along with cholesterol, polyunsaturated fatty acids (PUFA) play a role in determining membrane fluidity, and dietary sources of PUFA's can be incorporated into cell membranes, including T cell micro-domains, thus affecting signaling and cell functions (Fan et al., 2003; Switzer et al., 2004). Diets of free ranging whales may be more varied than in aquaria and it would be interesting to investigate the role of different diets on lymphocyte membrane composition and the effects of pressure.

This study provides the first evidence suggesting that anthropogenic activity which elicits a neuroendocrine response (e.g., boat noise) could impact the ability for beluga PBMC to function normally during diving either through the response itself, or by initiating changes in dive behavior. While small sample sizes limit the statistical power of the results, it is apparent that the relationship between the functional response of marine mammal immune cells to diving is complicated and more work needs to be done to better describe the conditions under which undesirable immune activity may occur. Such information is useful in trying to interpret growing reports of injuries in marine mammals that resemble dive related disease. Next steps of research should include *in vivo* measurements of dive related changes in immune function if possible (e.g., through behavioral training) in order to begin describing the combined effects of physiological adjustment and dive behavior.

This work follows on from that presented in Thompson and Romano (2015) which described changes in granulocyte activation and granulocyte and monocyte phagocytosis in response to *in vitro* pressure exposures. Here, the response of beluga lymphocytes and isolated PBMC to changes in pressure under different physiological conditions is presented. In both studies, some unexpected changes in immune function were measured following pressure exposures, including decreased phagocytosis, increased IL2R expression (lymphocyte activation) and decreased lymphocyte proliferation. Additionally, both studies found that increased neuroendocrine activity altered the response of beluga immune cells to pressure. While more work needs to be done to determine the implications of such changes, the results of this work provide evidence that different types of beluga immune cells may respond differently during dives, and that the response of cells can be altered by neuro-endocrine responses to various stimuli. Such information is necessary to create a physiological understanding of dive adaptation in immune cells and may prove useful in assessing the potential impact of anthropogenic activities on the health of marine mammal populations.

## Author contributions

LT contributed to all aspects of this work including project design, carrying out experimental assays, data analyses, and manuscript production. TR contributed to all aspects of this work including oversight of project design and experimental procedures, interpretation of data as well as manuscript editing.

## Funding

Funding for this work was provided by the Office of Naval Research award no. N00014-11-1-0437 and no. N00014-13-1-0768; the Mystic Aquarium; and the University of Connecticut Marine Science Department pre-doctoral fellowship awards. Additional funding was provided by the National Oceanic and Atmospheric Administrations (NOAA) Oceans and Human Health Initiative (OHHI) and Interdisciplinary Research and training Initiative on Coastal ecosystems and human Health (IRICH) program. Funding for field efforts in Bristol Bay was provided by Georgia Aquarium.

### Conflict of interest statement

The authors declare that the research was conducted in the absence of any commercial or financial relationships that could be construed as a potential conflict of interest. The reviewer CR and handling Editor declared their shared affiliation, and the handling Editor states that the process nevertheless met the standards of a fair and objective review
